# Retraction: Linc00472 suppresses breast cancer progression and enhances doxorubicin sensitivity through regulation of miR-141 and programmed cell death 4

**DOI:** 10.1039/d1ra90067f

**Published:** 2021-02-02

**Authors:** Laura Fisher

**Affiliations:** Royal Society of Chemistry Thomas Graham House, Science Park, Milton Road Cambridge CB4 0WF UK advances-rsc@rsc.org

## Abstract

Retraction of ‘Linc00472 suppresses breast cancer progression and enhances doxorubicin sensitivity through regulation of miR-141 and programmed cell death 4’ by Pengwei Lu *et al.*, *RSC Adv.*, 2018, **8**, 8455–8468, DOI: 10.1039/C8RA00296G

The Royal Society of Chemistry hereby wholly retracts this *RSC Advances* article due to concerns with the reliability of the data. The images in the article, and the raw data provided by the authors, were screened by an image integrity expert.

There are duplicating features among the ‘blue’ cells in the vector panel in Fig. 2D and the Linc00472 panel in Fig. 2E. The raw data provided by the authors for Fig. 2E showed clear evidence of manipulation.

The western blots in the article do not look genuine, and the raw data provided showed no background, which would not be expected if it was authentic raw data. Therefore, the raw data cannot be used to validate the published data.

Given the significance of the concerns about the validity of both the data in the article and the raw data provided by the authors, the findings presented in this paper are not reliable.

Yuanting Gu opposes the retraction. The other authors have been informed but have not responded to any correspondence regarding the retraction.

Signed: Laura Fisher, Executive Editor, *RSC Advances*

Date: 19^th^ January 2021

## Supplementary Material

